# A cost-effectiveness analysis of selective decontamination of the digestive tract in mechanically ventilated patients in the intensive care unit

**DOI:** 10.1016/j.ccrj.2026.100213

**Published:** 2026-09-09

**Authors:** Ashwani Kumar, Alamgir Kabir, John A. Myburgh, Anthony Delaney, Simon Finfer, Naomi E. Hammond, Sharon Micallef, Ian Seppelt, Balasubramanian Venkatesh, Colman Taylor, SuDDICU Australia Investigators and the Australian and New Zealand Intensive Care Society Clinical Trials Group

**Affiliations:** a The George Institute for Global Health, University of New South Wales, Sydney, Australia; b St George Hospital, Sydney, Australia; c Royal North Shore Hospital, Sydney, Australia; d Imperial College London, London, UK; e Nepean Hospital, Sydney, Australia; f Macquarie University, Sydney, Australia; g Wesley Hospital, Queensland, Australia

**Keywords:** Critical illness, Intensive Care Unit, Selective decontamination of the digestive tract, Cost-effectiveness

## Abstract

**Objective:**

To evaluate the 24-months cost-effectiveness of selective decontamination of the digestive tract (SDD) versus standard care in mechanically ventilated Intensive Care Unit (ICU) patients.

**Design:**

Within‑trial cost‑effectiveness analysis using linked administrative healthcare datasets.

**Setting:**

Nineteen Australian ICUs participating in the Australian Selective Decontamination of the Digestive Tract in the ICU (SuDDICU Australia) cluster crossover randomised trial (2018–2021).

**Participants:**

2845 adult patients enrolled from New South Wales, Australia with 24‑month follow‑up (SDD, n=1252; standard care, n=1593).

**Interventions:**

SDD plus standard care versus standard care alone.

**Main Outcome measures:**

All‑cause mortality at 24 months; life‑years gained; healthcare resource use; healthcare costs (Australian dollars [A$]) and cost-effectiveness expressed as incremental cost-effectiveness ratio (cost per life-year gained); subgroup analyses in acute brain injuries/conditions patients.

**Results:**

At 24 months, mortality (SDD: 521/1252 [41.6%] vs standard care: [697/1593] 43.8%; p=.25), and survival probabilities (hazard ratio 0.93, 95% confidence interval: 0.83-1.04; p=.22) did not differ between groups. SDD was associated with shorter median mechanical ventilation duration (4.0 vs 4.3 days) and lower mean non-SDD antibiotics use (11.7 vs 12.9) versus standard care. Mean per-patient healthcare costs were higher with SDD (A$294,529 vs A$268,493), yielding an incremental cost-effectiveness ratio of A$669,726 per life-year gained and ∼1% probability of SDD being cost-effectiveness at the Australian willingness-to-pay threshold. Incremental cost-effectiveness ratio was lower in traumatic brain injury patients (A$169,887 per life-year gained).

**Conclusion:**

In mechanically ventilated ICU patients, SDD use didn’t improve 24-month survival and healthcare costs were higher, resulting in low probability of cost-effectiveness within Australian healthcare context.

## 1 Introduction

Selective decontamination of the digestive tract (SDD) is an infection-control strategy designed to reduce mortality by preventing hospital-acquired infection in mechanically ventilated adult patients in the Intensive Care Unit (ICU).^1^ The principal aim of SDD is to prevent the development of hospital-acquired lower respiratory tract infections resulting from the overgrowth of aerobic Gram-negative bacteria and yeasts from the upper digestive tract.^2^^,^^3^ SDD usually involves the administration of topical nonabsorbable antimicrobials to the oropharynx and upper gastrointestinal tract, supplemented by a short course of intravenous antibiotics with a suitable antibiotic spectrum.

Recent clinical trials and meta-analyses have reported that SDD is associated with reduced incidence of ventilator-associated pneumonia and reduced in-hospital mortality compared to usual care.[4], [5], [6], [7] The trials did not demonstrate an increase in antimicrobial resistance, although these trials were conducted in low-antibiotic resistance settings.

Globally, the widespread adoption of SDD as a standard of care has been low due to uncertainty about longer-term changes in unit-based antimicrobial ecology, the lack of widespread commercial-grade SDD drug preparations, and whether SDD is a cost-effective intervention in critically ill patients.^8^^,^^9^

The Australian Selective Decontamination of the Digestive Tract in the Intensive Care Unit (SuDDICU Australia) trial^4^ was a randomised clinical trial to determine whether the use of SDD reduced hospital mortality in patients receiving invasive mechanical ventilation in the ICU compared to standard care alone. The trial reported that while the use of SDD did not result in a significant reduction in hospital mortality, the 95% confidence intervals (CIs) around the effect estimate included a clinically important benefit. Additionally, SDD use was associated with significant reduction in positive blood cultures and cultures with antibiotic-resistant organisms. Although the SuDDICU trial evaluated the clinical effectiveness of SDD use in the critically ill patients, the implications for healthcare resource use and costs remain uncertain.

Therefore, this prespecified cost-effectiveness analysis of the SuDDICU Australia trial was conducted from a healthcare payer perspective within the Australian healthcare context to determine 24-mo survival, healthcare resource use, and cost-effectiveness in participants receiving SDD plus standard care or standard care alone.

## 2 Methods

### 2.1 Trial design

The SuDDICU Australia trial was a crossover, cluster randomised clinical trial conducted in 19 ICUs in Australia between April 2018 and May 2021 that recruited 5982 participants, of which 2791 received SDD plus standard care and 3191 received standard care alone. The trial evaluated the effect of SDD on hospital mortality within 90 d after enrolment.^4^ Participating ICUs were randomly assigned to the SDD or standard care group for two alternating 12-mo periods, separated by a 3-mo interperiod gap. Patients in the SDD group received a 6-hourly application of an oral paste and administration of a gastric suspension containing colistin, tobramycin, and nystatin for the duration of mechanical ventilation plus a 4-d course of an intravenous antibiotic with a suitable antimicrobial spectrum. The trial was approved by a human research ethics committee with a waiver of individual patient informed consent.

For the SuDDICU cost-effectiveness analysis, the trial protocol and statistical analysis plan were prospectively developed (Online Resource 1). The analysis was conducted in the intention-to-treat population. Analyses were limited to patients enrolled in the state of New South Wales (NSW) of Australia, where healthcare resource use was available by linking the trial data with administrative health databases for the duration of 24 months after enrolment. Ethics approval for data linkage to administrative health records was obtained from the New South Wales Population and Health Services Research Ethics Committee (2020/ETH00148 dated 23 April 2024).

### 2.2 Data linkage

The data linkage period was from 1 January 2018 (first patient enrolled into the SuDDICU trial) until 30 June 2023 (25 mo after the last patient was enrolled). The follow-up period for the SuDDICU trial was censored at 90 d after enrolment. To capture 24-mo hospital-based healthcare resource use and costs, trial data were linked to administrative health datasets by the New South Wales (NSW) Centre for Health Record Linkage using probabilistic record linkage methods.^10^ Linked data included NSW Registry of Birth, Deaths and Marriages, Admitted Patient Data Collection, and Emergency Department Data Collection. Mortality data were obtained from the NSW Registry of Birth, Deaths and Marriages. Hospital admissions and length of stay were derived from Admitted Patient Data Collection, while Emergency Department presentations were obtained from the Emergency Department Data Collection.

### 2.3 Procedures and outcomes

#### 2.3.1 Primary clinical outcome

All-cause mortality was assessed at 24 mo after enrolment. Vital status (alive or dead) and date of death were obtained from the NSW Registry of Birth, Deaths and Marriages dataset.

#### 2.3.2 Health economic outcomes

Healthcare resource use outcomes included the duration of mechanical ventilation, duration of the index and subsequent ICU and hospital admissions, number of emergency department admissions, microbiology cultures, the number of SDD drug preparation kits, and non-SDD antibiotics. Daily doses of antibiotics were defined according to the World Health Organization Collaborating Centre for Drug Statistics Methodology.^11^

### 2.4 Healthcare resource use and cost estimation

Healthcare costs included index hospital admission, emergency department presentations, and hospital and ICU readmissions during the 24-mo period after enrolment. To avoid double counting, emergency department presentations were not separately costed as a substantial proportion resulted in hospital admissions and were therefore captured within hospital costs. Hospital costs were calculated using publicly available Australian government reimbursement figures taken from Australian Refined Diagnosis-Related Groups (version 6) for each episode of care.^12^ As the Australian Refined Diagnosis-Related Groups used in this study corresponded to 2012–2013, a factor of 1.3 was applied to adjust for inflation based on the comparison between 2024 and 2013 using the Reserve Bank of Australia inflation calculator.^13^

As this study was conducted in Australian ICUs, all costs are reported in Australian dollars (A$), where A$1.0 equated to US dollars (US$) $0.67 as on 30 June 2024.^14^

ICU costs were separately calculated using a fixed per-day cost of A$4375, based on national estimates, multiplied by the length of ICU stay.^15^ Emergency department costs were sourced from the New South Wales Emergency Department Data Collection that provides information about patient presentations to the emergency departments of public hospitals in NSW.

The cost-effectiveness analysis was conducted from the healthcare payer perspective of the Australian healthcare system. Australia has a universal healthcare system, in which the public hospital system provides access for services incurred.

To calculate cost-effectiveness, total hospital-related costs and life-years gained over 24 mo were determined. Incremental cost-effectiveness ratios providing the cost per life-year gained were calculated as a ratio between the difference in mean costs between the patients who received SDD and those who received standard care and the difference in mean life-years gained between those groups.

Sensitivity analysis was conducted by way of nonparametric bootstrapping with 10,000 replications using unrestricted random sampling,^16^^,^^17^ presented as cost-effectiveness planes to report the overall incremental cost per life-year gained. Cost-effectiveness acceptability curves were plotted to express the probability that the SDD would cost less than commonly used willingness-to-pay thresholds of A$100,000 (equivalent of US$67,000) per life-year gained at 24 mo in Australia. This threshold is based on empirical evidence and contemporary evidence from Australian funding decisions.[18], [19], [20]

In accordance with the prespecified statistical analysis plan, the cost-effectiveness analysis was also done in a subgroup of patients with acute brain injuries or conditions (traumatic brain injury, subarachnoid haemorrhage and stroke, and other brain injuries) following the publication of a post hoc analysis of the SuDDICU Australia trial that reported a lower mortality in this subgroup of patients associated with SDD.^21^

### 2.5 Statistical analysis

Baseline characteristics were summarised using summary statistics. Healthcare resource use and cost data were summarised using mean (standard deviation [SD]) and median (interquartile range [IQR]). Between-group comparisons were performed using t-test for symmetrically distributed continues variables, Wilcoxon rank-sum for asymmetrically distributed continuous variables, and chi-squared or Fisher’s exact (when expected cell count <5) tests were used for categorical variables. The probability of survival was assessed using Kaplan–Meier survival curve, and hazard ratio (HR) with 95% confidential interval (CI) were was estimated using Cox proportional-hazards model to compare survival probabilities between treatment arms. In a post- hoc analysis, restricted mean survival time (RMST) up to 720 d (24 mo) was estimated as the area under the Kaplan–Meier survival curve. The between-groups difference in RMST was calculated over this period with 95% CI estimated using nonparametric bootstrap resampling with 1000 replicates, recalculating RMST for each resample and deriving percentile-based CI from the bootstrap distribution. Cost-effectiveness analyses did not account for clustering by ICU, treatment sequence, or period effects and no adjustment for baseline covariates was done. Statistical significance was defined as a two-sided p value ≤ 0.05.

### 2.6 Trial registration

ACTRN12615000411549 and NCT02389036.

## 3 Results

Results are reported using the Consolidated Health Economic Evaluation Reporting Standards^22^ of Enhancing the Quality and Transparency of Health Research network and recommendations for economic evaluations of Intensive Care trials in Australia and New Zealand.^23^

### 3.1 Study population

**Fig. 1:**
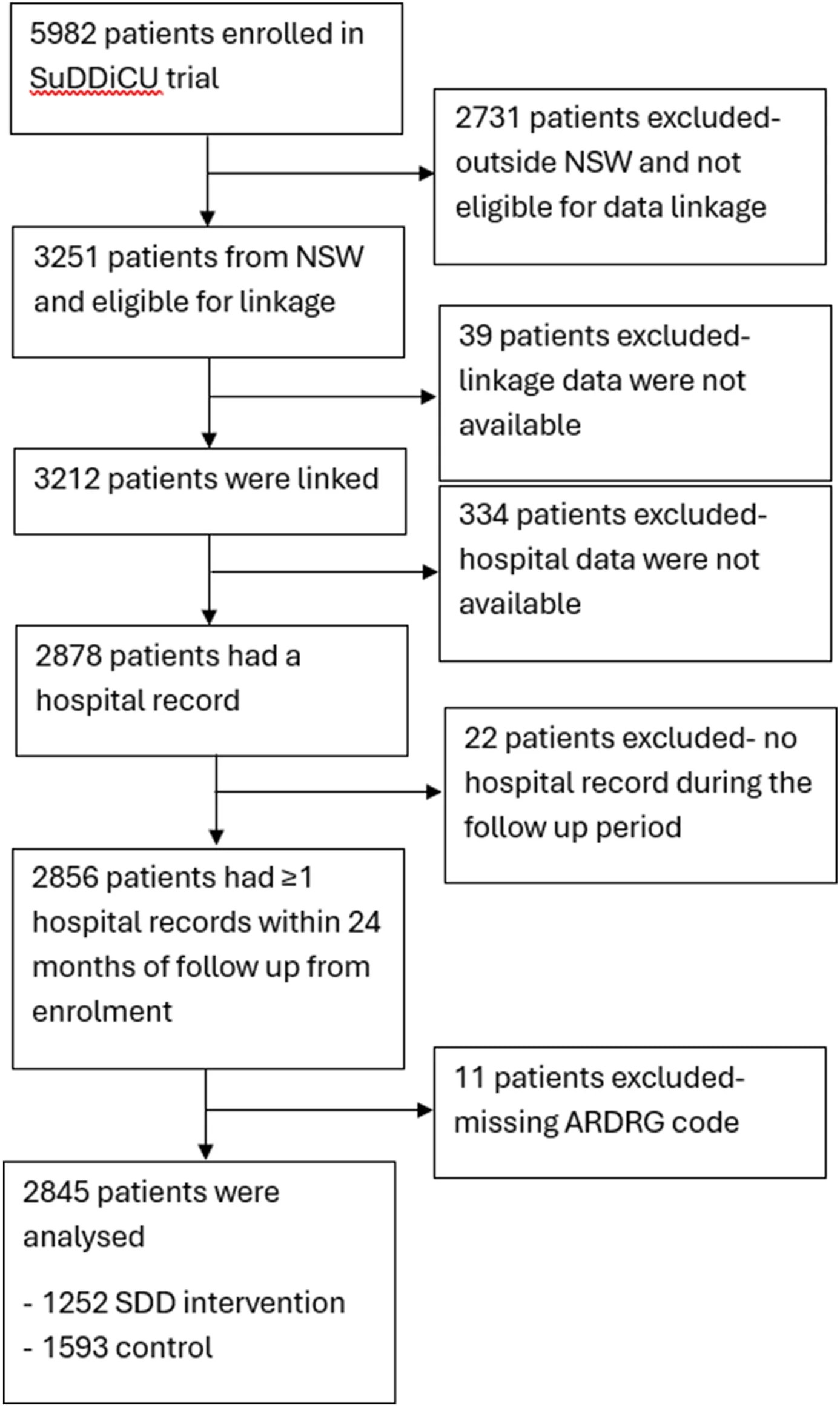
Consort diagram.

Of the 5982 participants enrolled in the SuDDICU Australia trial, 3251 (45.3%) were eligible for inclusion in the cost-effectiveness analysis. Among these, linked data were available for 2845 (1252 [44.0%] in the SDD group and 1593 [56.0%] in the standard care group, respectively) who were included in this analysis (Fig. 1).

### 3.2 Patient characteristics

Mean (SD) age was 59.2 (17.3) and 59.6 (17.0) years, and the proportion of females was 456/1252 (36.4%) vs 621/1593 (39.0%) in the SDD and standard care groups, respectively. Other baseline characteristics were similar between the SDD and standard care groups and consistent with that given in the main trial. Numerical differences were observed in Acute Physiology and Chronic Health Evaluation (APACHE-II) scores, time from ICU admission to enrolment, and receipt of preenrolment of intravenous antibiotics (eTable 1 in Online Resource 1).

### 3.3 Clinical outcomes

There was no significant difference in mortality rate at 24 mo between the SDD and standard care group (521/1252 [41·6%] vs 697/1593 [43·8%]; p = 0.25). Similarly, the 24-mo survival probabilities were not significantly different between groups (HR = 0.93, 95% CI: 0.83, 1.04; p = 0.22). In a post hoc analysis, RMST over 24 mo (720 d) was 19.61 d in the standard care group and 20.03 d in the SDD group (mean difference 0.43 d; 95% CI: −0.13, 0.96) (eFig. 1 in Online Resource 1).

### 3.4 Healthcare resource use

Healthcare resource use was similar between the SDD and standard care groups, except for a shorter duration of mechanical ventilation (median [IQR]: 4.0 [2.0–8.0] vs 4.3 [2.3–8.8]) d) and longer duration of ICU admission in the SDD group (median [IQR]: 8.6 [5.2–14.8] vs 8.1 [4.7–14.1] days). The mean (SD) defined daily dose of non-SDD antibiotics administered was significantly lower in the SDD group 11.7 (20.1) than the standard care group 12.9 (19.7); Table 1.

**Table 1:** Healthcare resource use among patients receiving SDD versus standard care over 24 mo.

Characteristic	Overall N = 2845	Standard care N = 1593	SDD N = 1252	*P* value
Duration of mechanical ventilation (d), median (IQR)	4·1 (2·1, 8·5)	4·3 (2·3, 8·8)	4·0 (2·0, 8·0)	<0.001
ICU length of stay (d), median (IQR)	8·3 (4·9, 14·3)	8·1 (4·7, 14·1)	8·6 (5·2, 14·8)	0.04
Index hospitalisation length of stay (d), median (IQR)	17·0 (10·0, 32·0)	17·0 (10·0, 31·0)	18·0 (10·0, 34·0)	0.08
Number of Emergency Department visits, mean (SD)	3·8 (6·0)	3·9 (6·1)	3·7 (5·9)	0.17
Number of hospital readmissions, mean (SD)	5·8 (24·9)	6·3 (27·0)	5·2 (21·8)	0.38
Length of stay during readmissions (d), median (IQR)	6·0 (0·0, 38·0)	5·0 (0·0, 38·0)	7·0 (0·0, 39·0)	0.17
Non-SDD antibiotic use (DDD), mean (SD)	12·4 (19·9)	12·9 (19·7)	11·7 (20·1)	<0.001
Number of SDD kits used^a^, mean (SD)	0·8 (1·3)	NA	1·7 (1·5)	

DDD = defined daily dose; SDD = selective decontamination of the digestive tract; SD = standard deviation; IQR = interquartile range; NA = not applicable; d = days.

a Each SDD kit included 20 doses of SDD oral paste and SDD gastric suspension.

Fewer patients had blood cultures (59.7% vs 66.4%) or respiratory cultures (9.3% vs 16.3%) collected in the SDD group compared to the standard care group; however, no statistical comparison was done. The proportions of patients with other types of cultures collected were comparable between groups (eTable 3 in Online Resource 1).

### 3.5 Cost-effectiveness outcomes

The SDD intervention costs represented 0.14% of the total healthcare cost. The mean per-patient total healthcare cost in the SDD group was significantly higher by A$26,036 than the standard care group (SDD: A$294,529 [SD 347,329] vs standard care: A$268,493 [SD 282,858]; p = 0.05). With a life-year gain of 0.04, this cost difference corresponds to an incremental cost-effectiveness ratio of A$669,726 per life-year gained. Mean costs were significantly higher in the SDD group for index hospitalisation (A$138,963 [SD 190,586]) vs A$124,644 [SD 157,404]; p = 0.02), index ICU admission (A$72,694 [SD 82,366] vs A$67,017 [SD 66,128]; p = 0.04), and intervention-related costs (A$400 [SD 315] vs A$57 [SD 76]; p<·001). Hospital readmission costs were similar (Table 2 and eTable 2 in Online Resource 1).

**Table 2:** Per-patient healthcare costs in overall, SDD, and standard care groups at 24 mo.

Characteristic	Overall (N = 2845)	Standard care (N = 1593)	SDD (N = 1252)	*P* value
SDD kit^a^				–
- Median (IQR)	0.00 (0, 185)	Not applicable	185 (185, 371	
- Mean (SD)	143 (243)	Not applicable	324 (276)	
Systemic antibiotics part of SDD^b^				–
- Median (IQR)	0 (0, 0)	Not applicable	0 (0, 10)	
- Mean (SD)	2 (5)	Not applicable	5 (7)	
Cost associated with delivery of SDD^c^				–
- Median (IQR)	0 (0, 0)	Not applicable	00 (0, 62)	
- Mean (SD)	13 (32)	Not applicable	29 (43)	
Microbiology cost^d^				<0.001
- Median (IQR)	31 (0, 64)	31 (0, 65)	30.8 (0.0, 34)	
- Mean (SD)	51 (73)	57 (76)	43 (67)	
**Total intervention cost (A)**				<0.001
- Median (IQR)	119 (31, 281)	31 (0, 65)	281 (216, 473)	
- Mean (SD)	208 (276)	57 (76)	400 (315)	
Intensive Care Unit admission cost^e^				0.04
- Median (IQR)	48,622 (28,885, 84,009)	47,490 (27,586, 82,547)	50,283 (30,353, 86,730)	
- Mean (SD)	69,516 (73,756)	67,017 (66,128)	72,694 (82,366)	
Index hospitalisation cost^f^				0.02
- Median (IQR)	78,522 (41,273, 157,044)	78,522 (39,445, 149,575)	83,757 (41,878, 167,513)	
- Mean (SD)	130,945 (172,908)	124,644 (157,404)	138,963 (190,586)	
Hospital readmission cost^f^				0.30
- Median (IQR)	11,845 (0, 83,892)	10,959 (0, 83,396)	12,794 (0, 83,952)	
- Mean (SD)	79,282 (165,278)	76,775 (155,463)	82,473 (176,993)	
**Total hospital cost (B)**				0.06
- Median (IQR)	183,489 (93,822, 342,601)	176,096 (91,717, 340,196)	192,487 (94,925, 343,794)	
- Mean (SD)	279,743 (312,972)	268,436 (282,824)	294,130 (347,157)	
**Total healthcare cost (A + B)**				0.05
- Median (IQR)	183,520 (93,938, 342,718)	176,126 (91,748, 340,289)	192,735 (95,144, 344,526)	
- Mean (SD)	279,951 (313,080)	268,493 (282,858)	294,530 (347,329)	

AR-DRG = Australian Refined Diagnosis-Related Groups; SD = standard deviation; SDD = selective decontamination of the digestive tract; IQR = interquartile range; mo = months.

All costs are in Australian dollars (A$) where 1.0 A$ equated to US dollar (US$) 0.67 as on 30 June 2024.

a Calculated based on the average number of SDD kits used per-patient, with the cost of each SDD kit taken as A$185.3.

b Defined as Cefotaxime 1g 6-hourly, or Ceftriaxone 1g daily, or Ciprofloxacin 400 mg 12-hourly. This was calculated using the mean number of doses for each of these antibiotics and their respective cost as A$3.5 per dose, A$3.2 per dose, and A$1.6 per dose, as listed on the Pharmaceutical Benefits Scheme, Australia (Source: https://www.pbs.gov.au/pbs/home).

c Calculated based on the assumption that it took 0·5 h by the nursing staff to deliver each SDD dose. Average hourly salary for the nursing staff was used as A$41·2 (Source: https://www.nswnma.asn.au/wp-content/uploads/2023/09/PHS-calculator-2023-07-01-1.pdf).

d Calculated using cost of individual culture listed by the Medicare Benefits Scheme in Australia (Source: https://www9.health.gov.au/mbs/search.cfm).

e Calculated using mean cost per-patient bed-day at Intensive Care Unit (ICU) of A$4375, as reported in the literature.

f Calculated by multiplying the total length of stay (same-day hospital discharges were counted as one full day) for each patient with the per-day hospital cost associated with the specific AR-DRG after adjusting for inflation (Source: https://www.aihw.gov.au/reports/hospitals/ar-drg-data-cubes/contents/summary).

**Fig. 2:**
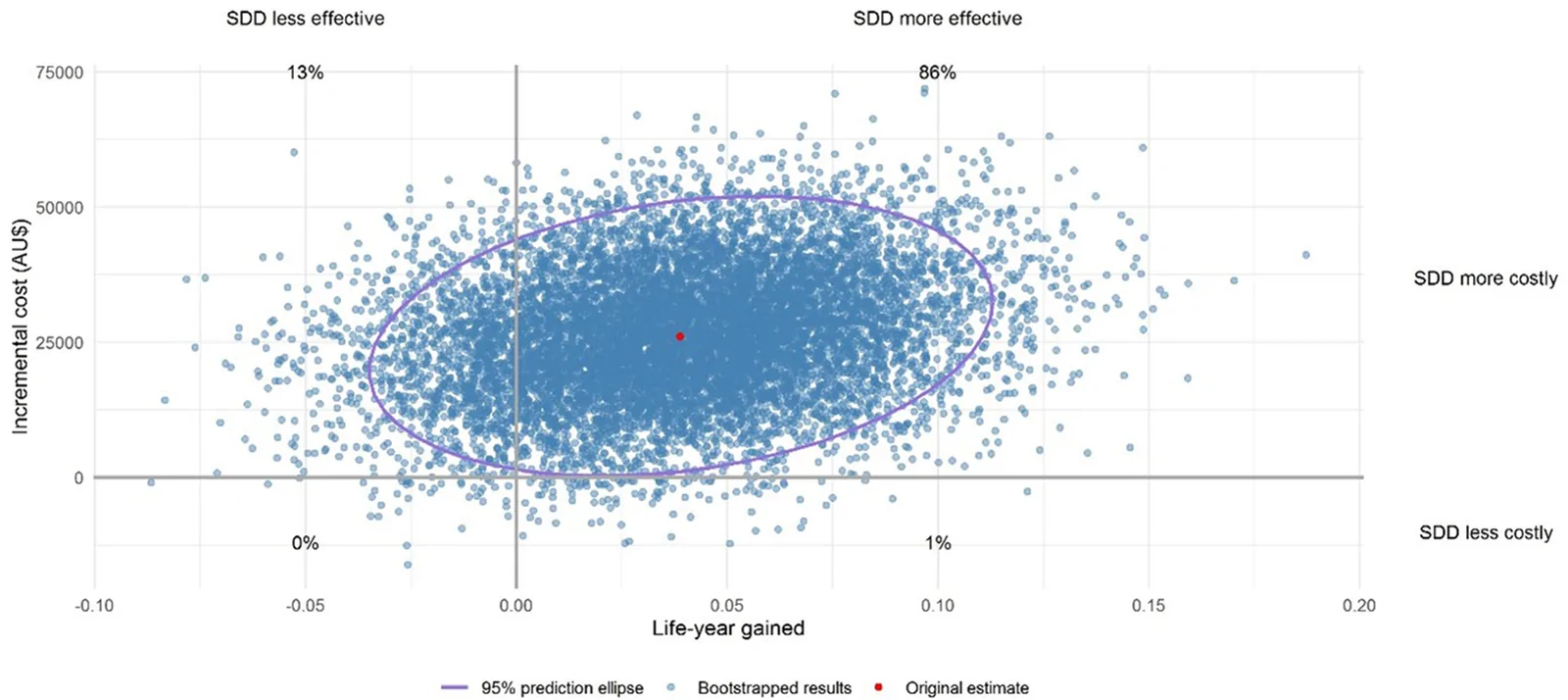
Cost-effectiveness plane of SDD versus standard care over 24 mo. A$ = Australian dollar; SDD = selective decontamination of the digestive tract; mo = months. Note: The horizontal axis represents the difference in life-years gained between SDD and standard care. The vertical axis represents the difference in cost between SDD and standard care. Each dot point represents a replication of the incremental cost and life-years gained between SDD and standard care derived from the bootstrapping procedure.

**Fig. 3:**
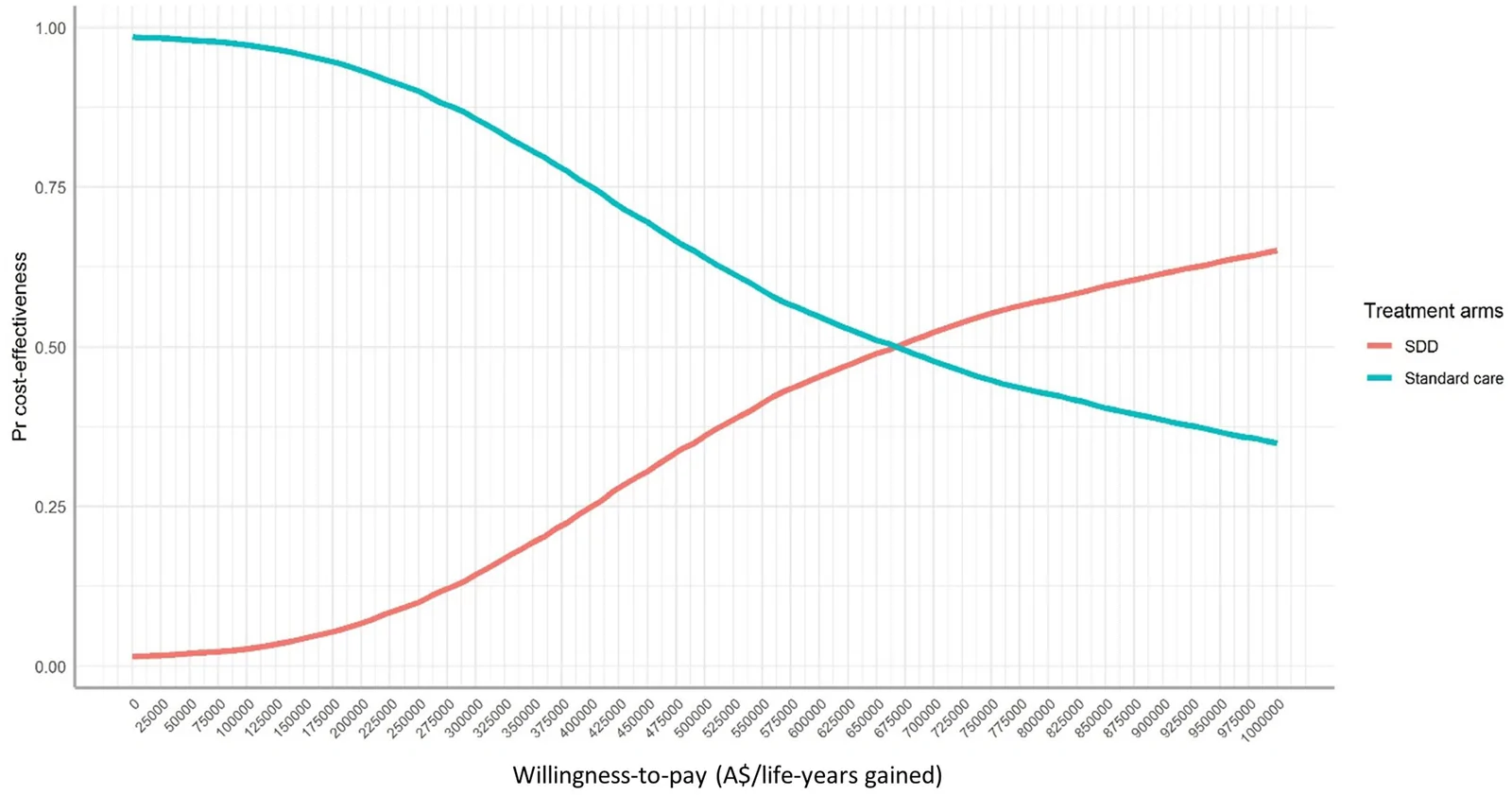
Cost-effectiveness acceptability curve of SDD versus standard care over 24 mo. A$ = Australian dollar; SDD = selective decontamination of the digestive tract; Pr = probability.

The cost-effectiveness plane showed that SDD was associated with higher healthcare costs in 99% of replications and increased life-years gained in 87% of replications that standard care (Fig. 2). The cost-effectiveness acceptability curve demonstrated a 1% probability of SDD being cost-effective at the Australian willingness-to-pay threshold of A$100,000 (equivalent to US$67,000) (Fig. 3).

### 3.6 Subgroup analysis

Over 24 mo, the incremental costs of SDD varied substantially across patient subgroups with acute brain injuries or conditions. Compared with the overall trial population, the incremental cost-effectiveness ratio was lower in patients with other brain injuries (incremental cost-effectiveness ratio A$34,142 per life-year gained; incremental cost, A$2899) and traumatic brain injury (incremental cost-effectiveness ratio A$169,887 per life-year gained; incremental cost, A$44,818). By contrast, patients with subarachnoid haemorrhage or stroke had the highest incremental cost-effectiveness ratio (A$696,439 per life-year gained; incremental cost, A$67,930). Patients without acute brain injury experienced a reduction in life-years (−0.02 life-years gained) despite incremental costs of A$19,865 (eTable 2 in Online Resource 1).

## 4 Discussion

In this economic evaluation of the SuDDICU Australia study, SDD was not significantly associated with improved 24-mo survival compared with standard care. Overall healthcare resource use was otherwise similar between groups. The index hospitalisation and ICU admission were the major cost drivers, with SDD intervention costs contributing only 0.14% of total healthcare costs. SDD was associated with higher total healthcare costs over 24 mo with incremental cost-effectiveness ratio of A$669,726 per life-year gained and about 1% probability of SDD being cost-effective at the Australian willingness-to-pay threshold.

The clinical effectiveness and implementation feasibility of SDD in ICU settings have been debated for decades, balancing potential survival benefits against antimicrobial stewardship considerations.[1], [2], [3], [4], [5], [6], [7], [8], [9] This study adds to the evidence base by providing a cost-effectiveness evaluation of SDD extending to 24 mo using data from a randomised clinical trial. Our findings differ from those of a previous individual patient data meta-analysis, which reported SDD to be cost-effective compared with selective oropharyngeal decontamination.^24^ Studies included in that meta-analysis were however limited to outcomes up to hospital discharge, whereas our study included linked healthcare use and survival data over a 24-mo period.

This study was embedded within a large, pragmatic randomised trial with standardised outcome assessment and a prespecified, economic analysis framework. The use of linked administrative data enabled comprehensive capture of resource use and costs across the index hospitalisation, ICU stay, and readmissions, with follow-up to 24 mo.

Approximately 50% of the SuDDICU Australia trial population were eligible for the cost-effectiveness analysis, which may limit the generalisability of these results to the overall Australian and similar international contexts. The use of the NSW death registry may have resulted in missed deaths among participants who died outside the state. This study focused on life-years gained rather than quality-adjusted life-years, thereby assessing health-related quality of life or functional recovery. The economic evaluation did not account for potential clustering by ICU, treatment sequence, or period effects. While substantial clustering of healthcare costs was not anticipated, this approach may have underestimated uncertainty if such effects were present. As the study was conducted in a low antimicrobial resistance environment, findings may not be generalisable to other health systems with higher antimicrobial resistance patterns.

From an Australian health system perspective, routine use of SDD in Australian ICUs cannot be considered to be cost-effective at the current willingness-to-pay threshold, given the incremental cost-effectiveness ratio exceeding this level and the low probability of cost-effectiveness.

Although the intervention cost was relatively low, the overall economic impact was largely driven by higher costs for index hospitalisation and readmissions in the SDD group. Health services planning should take this into account while considering any pilot implementation. Importantly, subgroup analyses showed heterogeneity in cost-effectiveness, with more favourable data in patients with acute brain injuries. This suggests that implementation in these subgroups may be warranted, acknowledging that these findings require further validation.

## 5 Future research

The heterogeneity observed across the acute brain injury subgroup warrants focused future research to determine whether tailored SDD strategies can achieve acceptable cost-effectiveness in specific patient populations. Future studies should incorporate quality-adjusted life-years and functional outcomes to capture patient-level metrics. In addition, further studies in diverse health systems, particularly those with higher antimicrobial resistance, are warranted to generate more generalisable data.

## 6 Conclusions

In participants enrolled in the SuDDICU trial, SDD did not significantly reduce longer-term mortality and was associated with increased healthcare costs over 24 mo compared to standard care. The resulting incremental cost-effectiveness ratio suggests that SDD is not cost-effective under current willingness-to-pay thresholds in Australia.

## Declaration of informed consent

Not applicable.

## Ethics approval

Ethics approval for data linkage to administrative health records was obtained from the New South Wales Population and Health Services Ethics Committee (2020/ETH00148 dated 23 April 2024).

## Data availability

Data will be shared in accordance with The George Institute for Global Health Data Access and Sharing Policy.

## Author contributions

**Myburgh**, **Taylor**, **Hammond**, **Seppelt**, **Finfer**, and **Kumar**: Concept and design. **Kumar**, **Taylor**, **Micallef**, and **Kabir**: Acquisition, analysis, or interpretation of data. **Kumar**, **Taylor**, **Myburgh**, and **Micallef**: Drafting of the manuscript. **Myburgh**, **Kumar**, **Taylor**, **Micallef**, **Seppelt**, **Hammond**, **Finfer**, **Venkatesh**, and **Delaney**: Critical revision of the manuscript for important intellectual content. **Kabir**: Statistical analysis. The authors were responsible for the study design, data collection, data analysis, data interpretation, and writing of the manuscript, independently of all funding sources.

## Funding

This work was supported by a 10.13039/501100000925National Health and Medical Research Council of Australia project grant (ID 1084244).

## Declaration of competing interest

Prof Myburgh reported receipt of grants from the National Health and Medical Research Council of Australia outside the submitted work. Prof Seppelt reported receipt of grants from the National Health and Medical Research Council of Australia outside the submitted work. Associate Prof Taylor is co-owner of a healthcare consultancy, HTANALYSTS, which undertakes paid consultancy work for various pharmaceutical and medical device companies as well as the Australian government. Prof Finfer reported receipt of nonfinancial support from Baxter Healthcare, grants from CSL Pty Ltd to his institution, and grants from Baxter Healthcare to his institution. No other disclosures were reported.

## References

[1] Bonten M.J., Kullberg B.J., van Dalen R., Girbes A.R., Hoepelman I.M., Hustinx W. (2000). Selective digestive decontamination in patients in intensive care. J Antimicrob Chemother.

[2] Rommes H., van Saene R., de la Cal M. (2021).

[3] Wittekamp B.H., Oostdijk E.A., Cuthbertson B.H., Brun-Buisson C., Bonten M.J. (2019). Selective decontamination of the digestive tract (SDD) in critically ill patients: a narrative review. Intensive Care Med.

[4] SuDDICU Investigators for the Australian and New Zealand Intensive Care Society Clinical Trials Group (2022). Effect of selective decontamination of the digestive tract on hospital mortality in critically ill patients receiving mechanical ventilation: a randomized clinical trial. JAMA.

[5] Hammond N.E., Myburgh J., Seppelt I., Garside T., Vlok R., Mahendran S. (2022). Association between selective decontamination of the digestive tract and in-hospital mortality in intensive care unit patients receiving mechanical ventilation: a systematic review and meta-analysis. JAMA.

[6] Plantinga N.L., de Smet A.M., Oostdijk E.A., de Jonge E., Camus C., Krueger W.A. (2018). Selective digestive and oropharyngeal decontamination in medical and surgical ICU patients: an individual patient data meta-analysis. Clin Microbiol Infect.

[7] Silvestri L., van Saene H.K., Zandstra D.F., Marshall J.C., Gregori D., Gullo A. (2010). Impact of selective decontamination of the digestive tract on multiple organ dysfunction syndrome: systematic review of randomized controlled trials. Crit Care Med.

[8] Hurley J.C. (2023). Establishing the safety of selective digestive decontamination within the ICU population: a bridge too far?. Trials.

[9] Hurley J.C. (2021). Selective digestive decontamination, a seemingly effective regimen with individual benefit or a flawed concept with population harm?. Crit Care.

[10] Centre for Health Record Linkage New South Wales, Australia. https://www.cherel.org.au.

[11] World Health Organization Defined daily dose (DDD). https://www.who.int/tools/atc-ddd-toolkit/about-ddd.

[12] Australian Institute of Health and Welfare Australian refined diagnosis-related groups (AR-DRG) data cubes. https://www.aihw.gov.au/reports/hospitals/ar-drg-data-cubes/contents/summary.

[13] Reserve Bank of Australia Inflation calculator. https://www.rba.gov.au/calculator.

[14] XE Currency Data Historical exchange rates. https://www.xe.com/currencytables.

[15] Hicks P., Huckson S., Fenney E., Leggett I., Pilcher D., Litton E. (2019). The financial cost of intensive care in Australia: a multicentre registry study. Med J Aust.

[16] Bland J.M., Altman D.G. (2015). Statistics notes: bootstrap resampling methods. BMJ.

[17] Briggs A.H., Gray A.M. (1999). Handling uncertainty when performing economic evaluation of healthcare interventions. Health Technol Assess.

[18] Australian Government Department of Health Pharmaceutical benefits scheme (PBS). https://www.pbs.gov.au.

[19] George B., Harris A., Mitchell A. (2001). Cost-effectiveness analysis and the consistency of decision making: evidence from pharmaceutical reimbursement in Australia (1991–1996). Pharmacoeconomics.

[20] Shiroiwa T., Sung Y.K., Fukuda T., Lang H.C., Bae S.C., Tsutani K. (2010). International survey on willingness-to-pay for one additional QALY gained: what is the threshold of cost effectiveness?. Health Econ.

[21] Young P.J., Devaux A., Li Q., Billot L., Davis J.S., Delaney A. (2024). Selective digestive tract decontamination in critically ill adults with acute brain injuries: a post hoc analysis of a randomized clinical trial. Intensive Care Med.

[22] Husereau D., Drummond M., Augustovski F., de Bekker-Grob E., Briggs A.H., Carswell C. (2022). Consolidated health economic evaluation reporting standards 2022 (CHEERS 2022) statement. MDM Policy Pract.

[23] Taylor C.B., Thompson K.J., Liew C., Rauniyar R. (2023). Economic evaluations for intensive care unit randomised clinical trials in Australia and New Zealand: practical recommendations for researchers. Aust Crit Care.

[24] van Hout D., Plantinga N.L., Bruijning-Verhagen P.C., Oostdijk E.A.N., de Smet A.M.G.A., de Wit G.A. (2019). Cost-effectiveness of selective digestive decontamination versus selective oropharyngeal decontamination in intensive care units with low levels of antimicrobial resistance: an individual patient data meta-analysis. BMJ Open.

