# Supplementary material for: A cost-effectiveness analysis of selective decontamination of the digestive tract in mechanically ventilated patients in the intensive care unit
Source: Crit Care Resusc. 2026 Sep 9;28(4):100213. doi: 10.1016/j.ccrj.2026.100213 (PMC13579672; doi:10.1016/j.ccrj.2026.100213)
Supplement: Supplementary file 1 [file mmc1.docx]

**A cost-effectiveness analysis of selective decontamination of the digestive tract in mechanically ventilated patients in the intensive care unit**

**Authors**

Ashwani Kumar^1^, Alamgir Kabir^1^, John A. Myburgh^1,2^, Anthony Delaney^1,3^, Simon Finfer^1,4^, Naomi E. Hammond^1,3^, Sharon Micallef^1^, Ian Seppelt^5,6^, Balasubramanian Venkatesh^1,7^ and Colman Taylor^1^ on behalf of the SuDDICU Australia Investigators and the Australian and New Zealand Intensive Care Society Clinical Trials Group.

1. The George Institute for Global Health and University of New South Wales, Sydney, Australia
2. St George Hospital, Sydney
3. Royal North Shore Hospital, Sydney, Australia
4. Imperial College London, UK
5. Nepean Hospital, Sydney, Australia
6. Macquarie University, Sydney, Australia
7. Wesley Hospital, Queensland, Australia

**Corresponding author:**

Dr Ashwani Kumar

The George Institute for Global Health

Level 8, Health Translation Hub

55 Botany Street Randwick, NSW 2031

Australia

Email: [akumar@georgeinstitute.org.au](mailto:akumar@georgeinstitute.org.au)

ORCID: 0000-0002-4787-7833.

Table of Contents

[Trial Sponsor, Committees, Funding Bodies and Endorsement	3](#_Toc220680932)

[Site Investigators	3](#_Toc220680933)

[Supplementary Methods – Administrative	3](#_Toc220680934)

[Supplementary Methods – Statistical Analysis Plan	5](#_Toc220680935)

[Supplementary Figures	12](#_Toc220680936)

[eFigure 1. Survival probabilities in SDD vs standard care group over 24 months	12](#_Toc220680937)

[Supplementary Tables	13](#_Toc220680938)

[eTable 1. Baseline characteristics of patients in overall, SDD and Standard care groups	13](#_Toc220680939)

[eTable 2: Life-years gained and incremental costs effectiveness ratio over 24 months in all patients and those with acute brain injury/conditions	14](#_Toc220680940)

[References	15](#_Toc220680941)

# Trial Sponsor, Committees, Funding Bodies and Endorsement

### Sponsor

The George Institute for Global Health, Australia.

### Writing Committee (for this manuscript)

Ashwani Kumar (Chair), Alamgir Kabir, John A. Myburgh, Sharon Micallef and Colman Taylor.

### Management Committee (SuDDICU Australia Trial)

John Myburgh (Chair), Laurent Billot, Brian Cuthbertson, Joshua Davis, Simon Finfer, Fiona Goodman, Naomi Hammond, Jon Iredell, Jennene Miller, Ian Seppelt, Colman Taylor.

### Funding bodies (SuDDICU Australia Trial)

The SuDDICU Australia trial was funded in Australia by a project grant from the National Health and Medical Research Council of Australia (Project Grant ID 1084244).

### Endorsement (for SuDDICU Trial)

The SuDDICU trial was endorsed by the Australian and New Zealand Intensive Care Society Clinical Trials Group.

# Site Investigators

Campbelltown Hospital: Deepak Bhonagiri, Monique Leijten, Sandhya Narayan, David Sanchez, Peta Saunders, Carli Sherriff

Gosford Hospital: Ellie Barker, Shelley Donovan, Katrina Ellis, Atul Gaur, Hannah Gibbons, Rebecca Gregory, Eloise Hair, Mary Keehan, Jess Naumoff, Elisha Turner

John Hunter Hospital: Gail Brinkerhoff, Dustin Bush, Federica Cazzola, Joshua Davis, Ken Havill, Paul Healey, Amber Poulter, Krishna Sunkara

Liverpool Hospital: Anders Aneman, Rachel Choit, Kelsey Dobell-Brown, Kairui Guo, Jillian Lee, Monique Leijten, Lien Lombardo, Zachariah Manalil, Jennene Miller, Jordan Rogers, Antony Stewart, Jana Yanga

Nepean Hospital: Rebecca Gresham, Julie Lowrey, Kristy Masters, Ian Seppelt, Christina Whitehead, Beverly Zaratan

Royal North Shore Hospital: Angela Ashelford, Frances Bass, Simon Finfer, Emily Fitzgerald, Oliver Flower, Naomi Hammond, Bernard Hudson, Pierre Janin, Elizabeth Limbrey, Sharon Mar, Anne O'Connor, Melissa Owen, Naomi Pallas, Julia Pilowsky, Veronica Roach, Elizabeth Ruse, Wade Stedman, Miyuki Tokumitsu, Elizabeth Yarad

St George Hospital: Deborah Inskip, Theresa Jacques, Adeline Kintono, Jennene Miller, Catherine Milner, John Myburgh, Rebecca Sidoli

Wollongong Hospital: Cartan Costello, Alan Davey-Quinn, Michael Davies, Ahmed Elgendy, Wenli Geng, Veerendra Jagarlamudi, Matthew Mac Partlin, Mahadev Patil, Adam Purdon, Martin Sterba

# Supplementary Methods – Administrative

### Trial Registration

The Selective Decontamination of the Digestive tract in Intensive Care Unit patients (SuDDICU) trial was registered on clinicaltrials.gov (NCT02389036) and the Australian and New Zealand Clinical Trials Registry (ACTRN12615000411549) before patient enrolment commenced.

### Data sharing

The SuDDICU investigators will share the trial data in a manner consistent with the Data Access and Sharing Policy of The George Institute for Global Health (available at https://www.georgeinstitute.org.au/data-sharing-policy) which governs appropriate and responsible sharing of, and access to, the Institute’s data for scientific research.

# Supplementary Methods – Statistical Analysis Plan

# Supplementary Figures

## eFigure 1. Survival probabilities in SDD vs standard care group over 24 months

Note: Patients not listed in the death database were considered to be alive. *Post hoc analysis

CI: Confidence Interval; SDD: Selective Decontamination of the Digestive tract; RMST: Restricted mean survival time; SDD: Selective Decontamination of Digestive tract.

# Supplementary Tables

## eTable 1. Baseline characteristics of patients in overall, SDD and Standard care groups

| Characteristic | Overall  N = 2,845 | Standard care  N = 1,593 | SDD  N = 1,252 |
| --- | --- | --- | --- |
| Age (in years) |  |  |  |
| Mean (SD) | 59·6 (17·0) | 59·9 (16·7) | 59·2 (17·3) |
| Sex |  |  |  |
| Female | 1077 (37·9%) | 621 (39·0%) | 456 (36·4%) |
| Male | 1768 (62·1%) | 972 (61·0%) | 796 (63·6%) |
| ICU admission source |  |  |  |
| Accident and Emergency Department | 1019 (35·8%) | 561 (35·2%) | 458 (36·6%) |
| Hospital floor (wards) | 506 (17·8%) | 290 (18·2%) | 216 (17·3%) |
| Transfer from another ICU | 222 (7·8%) | 122 (7·7%) | 100 (8·0%) |
| Transfer from another hospital | 281 (9·9%) | 154 (9·7%) | 127 (10·1%) |
| Admitted following emergency surgery | 664 (23·3%) | 372 (23·4%) | 292 (23·3%) |
| Admitted following ELECTIVE surgery | 153 (5·4%) | 94 (5·9%) | 59 (4·7%) |
| APACHE II SCORE (n=821 for control and n=744 for intervention)* |  |  |  |
| Median (IQR) | 20·0 (15·0, 26·0) | 20·0 (15·0, 25·0) | 21·0 (16·0, 27·0) |
| APACHE III SCORE (n=772 for control and n= 508 for intervention)* |  |  |  |
| Median (IQR) | 72·0 (51·0, 92·0) | 74·0 (53·0, 94·0) | 68·0 (49·0, 88·0) |
| Time from ICU admission to enrollment, days |  |  |  |
| Median (IQR) | 0·4 (0·0, 1·1) | 0·0 (0·0, 0·6) | 0·7 (0·3, 1·6) |
| APACHE diagnostic category: non-operative | 2028 (71·3%) | 1127 (70·7%) | 901 (72·0%) |
| Admission diagnosis of trauma | 404 (14·2%) | 220 (13·8%) | 184 (14·7%) |
| Diabetes | 664 (23·3%) | 380 (23·9%) | 284 (22·7%) |
| Systemic steroids | 318 (11·2%) | 164 (10·3%) | 154 (12·3%) |
| Immunosuppression | 269 (9·5%) | 152 (9·5%) | 117 (9·3%) |
| Received intravenous antibiotics at enrolment | 1990 (69·9%) | 1046 (65·7%) | 944 (75·4%) |
| Received intravenous antibiotics for >48 h prior enrolment | 559 (28·1%) | 262 (25·0%) | 297 (31·5%) |
| Use of oral chlorhexidine | 400 (14·1%) | 127 (8·0%) | 273 (21·8%) |

APACHE = Acute Physiology and Chronic Health Evaluation, DDD: Defined Daily Doses, IQR: Inter Quartile Range; SDD: Selective Decontamination of the Digestive tract; SD: Standard Deviation; IQR: Inter Quartile Range, ICU: Intensive Care Unit

*Severity of illness was measured using the APACHE II method for some patients and the APACHE III method for others; therefore, we presented the results separately for each method. APACHE scores range from 0 to 71 (APACHE II) or 0 to 299 (APACHE III), with higher scores indicating an increased risk of death.

## eTable 2: Life-years gained and incremental costs effectiveness ratio over 24 months in all patients and those with acute brain injury/conditions

| **Population subgroup** | **N** | **Cost (A$) difference** | **Life-years gained** | **ICER (costs (A$)/LYG)** |
| --- | --- | --- | --- | --- |
| Overall trial population | 2848 | 26,036 | 0·04 | 669,726 |
| Patients with traumatic brain injury | 244 | 44,818 | 0·26 | 169,887 |
| Patients with subarachnoid hemorrhage and stroke | 285 | 67,930 | 0·10 | 696,439 |
| Patients with other brain injury | 472 | 2,899 | 0·08 | 34,142 |
| Patients without acute brain injury | 1847 | 19,865 | -0·02 | - |

A$: Australian dollar; ICER, Incremental Costs Effectiveness Ratio; LYG: Life-years gained

## eTable 3: Antibiotic use and microbiology culture collection in overall, SDD and standard care group

| Characteristic | Overall  N = 2,845 | Standard care  N = 1,593 | SDD  N = 1,252 |
| --- | --- | --- | --- |
| Number of doses used of systemic antibiotics part of SDD, Mean (SD) |  |  |  |
| - Cefotaxime 1g 6 hourly | 0.1 (0.9) | NA | 0.1 (1.3) |
| - Ceftriaxone 1g 6 hourly | 0.5 (1.3) | NA | 1.2 (1.7) |
| - Ciprofloxacin 400 mg 12 hourly | 0.0 (0.4) | NA | 0.0 (0.5) |
| Number of non-SDD antibiotic use (DDD*), Mean (SD) | 12.4 (19.9) | 12.9 (19.7) | 11.7 (20.1) |
| - Number of patients with microbiology cultures obtained, N (%) |  |  |  |
| - Blood culture | 1,805 (63.4%) | 1,057 (66.4%) | 748 (59.7%) |
| - CSF/Brain culture | 14 (0.5%) | 6 (0.4%) | 8 (0.6%) |
| - Gastrointestinal culture | 10 (0.4%) | 3 (0.2%) | 7 (0.6%) |
| - Peritoneal fluid culture | 13 (0.5%) | 9 (0.6%) | 4 (0.3%) |
| - Pleural fluid culture | 17 (0.6%) | 10 (0.6%) | 7 (0.6%) |
| - Respiratory culture | 355 (13.2%) | 243 (16.3%) | 112 (9.3%) |
| - Urine/Urinary Tract culture | 88 (3.1%) | 53 (3.4%) | 35 (2.8%) |
| - Vascular catheter culture | 47 (1.7%) | 28 (1.8%) | 19 (1.5%) |
| - Wound/Skin culture | 89 (3.2%) | 60 (3.8%) | 29 (2.4%) |
| - Nasal/throat swab culture | 98 (3.4%) | 61 (3.8%) | 37 (3.0%) |
| - Perineal/rectal swab culture | 184 (6.5%) | 103 (6.5%) | 81 (6.5%) |
| - Deep surgical specimen culture | 20 (0.7%) | 10 (0.6%) | 10 (0.8%) |
| - Any microbiology culture | 1,985 (69.8%) | 1,167 (73.3%) | 818 (65.3%) |

DDD: Defined Daily Dose; NA: Not applicable; SDD: Selective Decontamination of the Digestive tract; SD: Standard Deviation.

*DDD is the assumed average maintenance dose per day for a drug used for its main indication in adults (Source: <https://www.who.int/tools/atc-ddd-toolkit/about-ddd>)

# References

1. Wittekamp BH, Oostdijk EA, Cuthbertson BH, Brun-Buisson C, Bonten MJ. Selective decontamination of the digestive tract (SDD) in critically ill patients: a narrative review. *Intensive Care Med*. 2019;46(2):343–349. doi: [10.1007/s00134-019-05883-9](https://doi.org/10.1007/s00134-019-05883-9)
2. de Smet AM, Kluytmans JA, Cooper BS, et al. Decontamination of the digestive tract and oropharynx in ICU patients. *N Engl J Med*. 2009;360:20‑31. DOI: 10.1056/NEJMoa0800394
3. The SuDDICU Investigators for the Australian New Zealand Intensive Care Society Clinical Trials Group. Effect of Selective Decontamination of the Digestive Tract on Hospital Mortality in Critically Ill Patients Receiving Mechanical Ventilation: A Randomized Clinical Trial. *JAMA*. 2022;328(19):1911–1921. doi:10.1001/jama.2022.17927
4. Billot L, Cuthbertson B, Gordon A, et al. Protocol summary and statistical analysis plan for the Selective Decontamination of the Digestive Tract in Intensive Care Unit Patients (SuDDICU) crossover, cluster randomised controlled trial. *Crit Care Resusc.* 2021;23(2):183‑193. https://doi.org/10.51893/2021.2.oa5
5. Pietropaoli AP, Glance LG, Oakes D, Fisher SG. Gender differences in mortality in patients with severe sepsis or septic shock. *Gender Med*. 2010;7(5):422-37. https://doi.org/10.1016/j.genm.2010.09.005
6. Public Health System Nurses’ & Midwives’ (State) Award, NSW Australia, 2023. <https://www.nswnma.asn.au/wp-content/uploads/2023/09/PHS-calculator-2023-07-01-1.pdf>
7. Australian Government Department of Health, Pharmaceutical Benefit Scheme. Available at: [<https://www.pbs.gov.au/pbs/home>]
8. Thompson KJ, Young PJ, Venkatesh B, et al. Long-term costs and cost-effectiveness of adjunctive corticosteroids for patients with septic shock in New Zealand. *Aust Crit Car*e. 2022 May 1;35(3):241-50. https://doi.org/10.1016/j.aucc.2021.05.006
9. Australian Government Department of Health. Round 14 (2009-10) National Public Cost Weight Tables - version 6.0x and version 5.2 2012 [Available from: <http://www.health.gov.au/internet/main/publishing.nsf/Content/Round_14-cost-reports>]
10. Hicks P, Huckson S, Fenney E, Leggett I, Pilcher D, Litton E. The financial cost of intensive care in Australia: a multicentre registry study. *Med J Aust*. 2019;211(7):324–5. doi: 10.5694/mja2.50309
11. Briggs AH, Gray AM. Handling uncertainty when performing economic evaluation of healthcare interventions. *Health Technol Assess*. 1999;3(2):1–134. <https://doi.org/10.3310/hta3020>
12. Bland JM, Altman DG. Statistics notes: bootstrap resampling methods. *BMJ*. 2015;350:h2622. doi: <https://doi.org/10.1136/bmj.h2622>
13. Shiroiwa T, Sung YK, Fukuda T, Lang HC, Bae SC, Tsutani K. International survey on willingness-to-pay (WTP) for one additional QALY gained: what is the threshold of cost effectiveness? *Health Econ*. 2010;19(4):422-37. https://doi.org/10.1002/hec.1481
14. Higgins AM, Harris AH. Health economic methods: cost-minimization, cost-effectiveness, cost-utility, and cost-benefit evaluations. *Crit Care Clin.* 2012;28(1):11-24. https://doi.org/10.1016/j.ccc.2011.10.002
